# Trastuzumab beyond progression in HER2-positive advanced breast cancer: The Royal Marsden experience

**DOI:** 10.1038/bjc.2011.138

**Published:** 2011-04-26

**Authors:** T Waddell, A Kotsori, A Constantinidou, N Yousaf, S Ashley, M Parton, M Allen, N Starling, P Papadopoulos, M O'Brien, I Smith, S Johnston

**Affiliations:** 1 Breast Unit Royal Marsden Hospital, Institute of Cancer Research, Fulham Road, London SW3 6JJ, UK; 2 Statistics, Royal Marsden Hospital, Fulham Road, London SW3 6JJ, UK

**Keywords:** trastuzumab, HER2 positive, metastatic breast cancer

## Abstract

**Background:**

Recent UK clinical guidance advises against continuing trastuzumab (T) beyond disease progression (PD) in the absence of brain metastases in patients with HER-2 positive (+ve) advanced breast cancer .We have retrospectively evaluated the outcome of patients with HER-2+ve locally advanced (LA) or metastatic breast cancer (MBC) who continued T beyond PD, treated in our unit.

**Methods:**

All HER-2+ve patients on our prospectively maintained database with LA or MBC who received T beyond PD after adjuvant or one line of T for advanced disease were assessed for response and outcome. From the timepoint of T continuation beyond PD, we calculated the overall disease control rate, time to progression (TTP), and overall survival (OS).

**Results:**

One hundred and fourteen patients with HER-2+ve LA or MBC treated with T beyond PD were identified. The main site of disease was visceral_in 84 (74%) patients. Seventy-six (66%) had one line of chemotherapy before continuation of T beyond PD and 21 (19%) had two or more. Post-progression, 66 (58%) received T combined with chemotherapy. Of the 93 (82%) patients with documented clinical or radiological response evaluation, 67 (59%) were considered as having stable disease or better. The median TTP was 24 weeks (95% CI: 21–28) and the median OS was 19 months (95% CI: 12–24).

**Conclusion:**

Our results from an unselected group of patients provide additional evidence that continuation of T beyond PD is of clinical benefit.

Development of resistance to treatment remains an important problem in HER2-positive breast cancer. At the point of disease progression (PD) on trastuzumab, there currently remains debate as to whether it should be continued alongside a change in other systemic therapy, switched/added to an alternative anti-HER2-targeted agent, or discontinued altogether. In preclinical studies, it was noted that continuation of trastuzumab had some ongoing effect against cell proliferation, with rapid tumour re-growth observed when it was subsequently withdrawn (Pietras *et al*, 1998; Pegram *et al*, 2004; Fujimoto-Ouchi *et al*, 2010). Randomised clinical data to support this preclinical work has previously been lacking, with evidence generally coming from retrospective analyses (Stemmler *et al*, 2005; Bartsch *et al*, 2007; Menard *et al*, 2008).

However, a recent prospective randomised phase III study (GBG26) showed benefit for trastuzumab continuation alongside capecitabine compared with capecitabine alone. Continuation was associated with a statistically significant improvement in time to progression (TTP), overall response rate (ORR), and a trend towards improved overall survival (OS) (Von Minckwitz *et al*, 2009). A second study (EGF100151) has looked at the possibility of switching at PD from trastuzumab to an alternative anti-HER2-targeted therapy, lapatinib. Women with HER2-positive breast cancers, which had progressed following treatment with anthracyclines, taxanes, and trastuzumab, were randomised to either capecitabine alone or capecitabine plus lapatinib with the primary end point defined as progression-free survival (PFS). The results showed statistically significant superiority of the combination therapy arm in terms of PFS, and a trend towards improved survival (Geyer *et al*, 2006).

Furthermore, results from a recent randomised phase II study (EGF104900) of lapatinib with or without trastuzumab, in a heavily trastuzumab pretreated population, showed that the combination performed better than the single-agent approach. The authors concluded that dual HER2 blockade is effective and offers the option of treatment without chemotherapy and its side effects (Blackwell *et al*, 2010).

Despite a growing body of evidence to support the continuation of trastuzumab at PD, NICE guidance in the UK states that ‘For patients who are receiving treatment with trastuzumab for advanced breast cancer, discontinue treatment with trastuzumab at the time of disease progression outside the central nervous system. Do not discontinue trastuzumab if disease progression is within the central nervous system alone’ (NICE Guidance, 2009).

Practising oncologists in the United Kingdom have therefore found that they can no longer obtain funding for trastuzumab beyond progression, a practice that was previously commonplace. In contrast, outside of the United Kingdom, the international breast cancer community continue to prescribe anti-HER2-targeted therapies alongside multiple lines of systemic treatment in this patient population.

On this ground, we considered it timely to assess retrospectively the clinical efficacy and safety of our practice to continue trastuzumab beyond progression.

## Patients And Methods

The study population comprised patients with metastatic or locally advanced HER2-positive breast cancer who continued trastuzumab beyond PD at the Royal Marsden Hospital between January 2001 and December 2008. All patients who progressed after one line of trastuzumab for advanced disease were included regardless of the number of previous non-trastuzumab therapies in the metastatic setting. Those who had received adjuvant trastuzumab were also eligible if they had relapsed during or within 12 weeks of completing adjuvant trastuzumab, and subsequently continued it into the advanced-disease setting.

Eligible patients with HER2-positive disease based on overexpression as detected by immunohistochemistry (3+ staining) or amplification by fluorescent *in situ* hybridisation (FISH) were identified from a prospectively maintained Royal Marsden Hospital breast research database, and from an electronic hospital pharmacy database of all dispensed doses of trastuzumab.

Relevant data were subsequently collected from individual electronic patient records. These included demographic data, information regarding the date of initial breast cancer diagnosis, date of metastatic or locally advanced disease, sites of metastases, ER/PgR status, and details of all previous and subsequent lines of therapy received.

Information was collected on the date trastuzumab was started, details of concurrent chemotherapy/hormone therapies, date of first documented progressive disease, changes to concurrent therapy at that point, and date of next progression after trastuzumab continuation.

Response data were collected from imaging reports where available and serial clinical assessments.

Discontinuation of trastuzumab due to cardiac toxicity including reduced left ventricular ejection fraction was also recorded from case records.

### Study end points

The primary end point of the study was TTP from the timepoint of trastuzumab continuation after PD. Secondary end points were overall disease control rate defined as patients achieving clinically or radiologically stable disease (SD) for ⩾3 months or better and OS.

In addition to our overall analysis, we also performed a subgroup analysis of those patients whose initial trastuzumab therapy was either in the first-line metastatic setting or had relapsed during, or within 12 weeks of completing adjuvant trastuzumab, and subsequently continued it into the advanced-disease setting (a population comparable to that of the GBG26 study).

### Statistical analysis

Time to progression on continuation of trastuzumab beyond progression was calculated by the Kaplan–Meier method and was measured from the date of first progression on trastuzumab until the next PD in any site. Patients were censored at last follow-up or at death in the absence of PD.

Overall survival was measured from the date of first progression until death from any cause and was censored at last follow-up.

A Cox proportional hazards model was applied to adjust for prognostic factors that influenced TTP, including age, ER/PgR status, sites of disease, adjuvant chemotherapy *vs* not, previous lines of chemotherapy for metastatic disease, and type of treatment combined with trastuzumab for metastatic disease (initial and beyond progression).

The study proposal was reviewed and approved by our Institutional Audit Committee.

## Results

### Patient demographics

**Figure 1:**
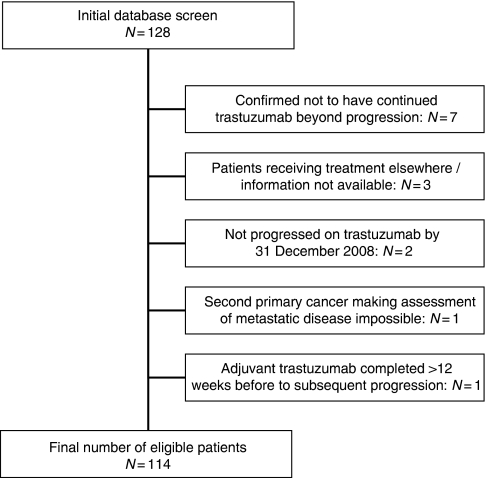
Identification of eligible patients.

Through our initial database screening, 128 patients were identified who appeared eligible for inclusion in the study. However, 14 of these patients were subsequently excluded from the data collection (see Figure 1) leaving a total of 114 patients. At the time of analysis, 35 patients (31%) were still alive with a median follow-up of 20 months. The main site of disease was visceral in 84 patients (74%), including 37 patients (32%) with central nervous system involvement. Twenty-six patients (23%) developed brain metastases while on first-line trastuzumab and continued trastuzumab beyond progression in the brain. Thirty patients (26%) had soft tissue and/or bone metastases only. Fifty-nine patients (52%) had received adjuvant chemotherapy, whereas only 13 (11%) had received adjuvant trastuzumab. Other baseline information and previous and subsequent lines of therapy are outlined in Table 1.

At the point of continuing trastuzumab beyond PD, 12 patients (11%) received this in combination with taxanes, 32 patients (28%) with capecitabine, and 22 patients (19%) with vinorelbine. Another four patients (4%) continued trastuzumab alongside endocrine therapy and 44 patients (38%) continued trastuzumab alone. Of these 44 patients, 29 (25%) received also some form of radiotherapy. All but two patients of the 26 who developed brain metastases while on first-line trastuzumab received brain radiotherapy alongside continuation of trastuzumab.

Seven (6%) patients received lapatinib as a subsequent line of therapy following the second progression on trastuzumab.

Eighty-one (71%) patients were found to match the GBG26 study population and their demographics and previous and subsequent therapies are shown in Table 2.

### Outcome

The median TTP for all patients was 24 weeks (95% CI: 21–28 weeks) and the median OS was 19 months (95% CI: 12–24 months). The median TTP did not differ (TTP: 24 weeks; 95% CI: 16–32) for the patients who developed brain metastases and continued trastuzumab beyond progression in the brain.

No prognostic factors were identified to predict for longer TTP.

Information regarding response after continuation of trastuzumab beyond PD was not available for 21 (18%) of the patients. Of the 93 patients (82%) with documented clinical (*n*=16) or radiological (*n*=77) response evaluation (Table 3), 67 (59%) were considered to have SD or better and 26 (23%) as having PD. It was not possible to differentiate between partial response and SD as this was a retrospective study and scan reporting was not always performed according to the RECIST criteria.

In the 81 patient subgroup analysis, overall disease control was achieved in 50 (61%) patients, the median TTP was 25 weeks (95% CI: 18–33 weeks), and the median OS was 22 months (95% CI: 17–27 months) (Table 4).

Only six (5%) patients overall had to discontinue trastuzumab secondary to decline in LVEF. No cardiac deaths were observed.

## Discussion

The findings of this study support a growing body of evidence that is now consistently showing improved response rates and TTP when trastuzumab is continued beyond PD in HER2-positive advanced breast cancer (Stemmler *et al*, 2005; Bartsch *et al*, 2007; Fabi *et al*, 2008; Von Minckwitz *et al*, 2009; Blackwell *et al*, 2010). We recognise that our study is limited by its retrospective nature and the lack of a comparable control group who did not continue trastuzumab at progression. Nevertheless, in this unselected group of patients, many of whom had been heavily pretreated, continuation of trastuzumab was associated with a clinically meaningful median TTP of 24 weeks.

Direct comparison of this study with other studies cannot be made. However, our results seem to compare favourably with the capecitabine-alone arm of the EGF100151 study (median TTP 4.4 months), (Geyer *et al*, 2006), suggesting efficacy of continued trastuzumab in our study population.

In the GBG26 study, the results are generally superior with the capecitabine-alone arm having a median TTP of 5.6 and the combination therapy arm having a median TTP of 8.2 months (Von Minckwitz *et al*, 2009). This difference likely reflects that eligible patients in the German study were only permitted to have one previous line of chemotherapy. In addition, in our study population many patients continued trastuzumab alongside palliative radiotherapy or change in hormone therapy, rather than with chemotherapy. It is worth noting here that it has been previously shown that trastuzumab does have particular synergy when combined with chemotherapy (Arteaga *et al*, 1994; Pegram *et al*, 1998, 1999).

Our results are entirely concordant with other retrospective analyses of trastuzumab continuation in this patient population (Stemmler *et al*, 2005; Bartsch *et al*, 2007; Fabi *et al*, 2008; Menard *et al*, 2008; Extra *et al*, 2010), including patients who developed brain metastases while on trastuzumab and continued trastuzumab beyond progression in the brain (Park *et al*, 2009). These studies share the common limitations of a retrospective approach. However, it seems highly unlikely that there will be further prospective, randomised studies examining the specific role of trastuzumab combined with second-line chemotherapy for women who have progressed on trastuzumab.

The international oncological community has already adopted the practice of continuing trastuzumab beyond PD. In contrast, practising oncologists in the UK are now finding that they can no longer obtain funding for trastuzumab beyond progression because of the NICE recommendation, which was based on the absence of published, level 1 evidence showing an improved OS associated with continuation of trastuzumab.

Further studies with novel anti-HER-2-targeted therapies, such as pertuzumab, TDM-1, and Neratinib, continue to explore the value of multiple lines of anti-HER2 therapies in this subpopulation of breast cancer patients (Baselga *et al*, 2010; Burstein *et al*, 2010; Burris *et al*, 2011). However, until these novel therapies establish their role, continuation of trastuzumab beyond progression should remain an option in the management of patients with HER2-positive advanced breast cancer. Furthermore, NICE should consider re-evaluating its current recommendation to ensure that breast cancer practice in the UK does not become significantly discordant with both the published evidence and the international oncological community.

In conclusion, our study supports the positive results and good safety data shown in previous studies. Continuation of trastuzumab beyond PD in women with HER2-positive advanced breast cancer is of meaningful clinical benefit and should remain a treatment option for this patient population.

## Figures and Tables

**Table 1:** Clinical characteristics of the 114 patients with HER2+ve locally advanced or MBC

**Median age (range)**	**56 (28–85)**		
**Hormone receptor status**	**+ve (%)**	**−ve (%)**	**nk (%)**
ER	58 (51)	56 (49)	—
PgR	17 (15)	70 (61)	27 (24)
			
**Metastatic site**	**Number of patients (%)**		
CNS	37 (32)		
Liver	61 (53.5)		
Lung	35 (31)		
Bone	66 (58)		
Soft tissue	31 (27)		
Nodes	42 (37)		
Any visceral/CNS	84 (74)		
Non-visceral	30 (26)		
	
*Adjuvant chemotherapy*	59 (52)		
Anthracycline based	39 (34)		
Taxane based	12 (11)		
Other	8 (7)		
	
Adjuvant trastuzumab	13 (11)		
	
*Number of previous lines of chemotherapy (before trastuzumab beyond progression)*			
0	17 (15)		
1	76 (66)		
2	18 (16)		
3	3 (3)		
	
*Previous therapy with trastuzumab*			
Trastuzumab alone	20 (18)		
Trastuzumab plus chemotherapy	86 (75)		
Anthracycline based	4 (4)		
Taxane based	53 (46)		
Vinorelbine	22 (19)		
Capecitabine	7 (6)		
Trastuzumab plus endocrine therapy	8 (7)		
	
*Duration of previous trastuzumab (weeks)*			
Median (range)	39 weeks (3 weeks–38 months)		
<12	11		
12–24	21		
>24	82		
	
*Treatment at progression*			
Trastuzumab alone	15 (13)		
Trastuzumab plus chemotherapy	66 (58)		
Taxane based	12 (11)		
Capecitabine	32 (28)		
Vinorelbine	22 (19)		
Trastuzumab plus endocrine therapy	4 (4)		
Trastuzumab plus radiotherapy	29 (25)		

Abbreviations: CNS=central nervous system; ER=oestrogen receptor; HER2=human epidermal growth factor receptor 2; MBC=metastatic breast cancer; PgR=progesterone receptor.

**Table 2:** Clinical characteristics of the 81 patients with HER2+ve locally advanced or MBC (same patient inclusion criteria as in the GBG26 study)

**Median age (range)**	**55 (29–84)**		
**Hormone receptor status**	**+ve (%)**	**−ve (%)**	**nk (%)**
ER	38 (47)	43 (53)	—
PgR	11 (13.5)	53 (65.5)	17 (21)
			
**Metastatic site**	**Number of patients (%)**		
CNS	28 (34.5)		
Liver	42 (52)		
Lung	24 (30)		
Bone	48 (59)		
Soft tissue	18 (22)		
Nodes	30 (37)		
Any visceral/CNS	63 (78)		
Non-visceral	18 (22)		
	
*Adjuvant chemotherapy*	59 (73)		
Anthracycline based	39 (48)		
Taxane based	12 (15)		
Other	8 (10)		
	
Adjuvant trastuzumab	7 (9)		
	
*Previous therapy with trastuzumab*			
Trastuzumab alone	11 (13.5)		
Trastuzumab plus chemotherapy	66 (81.5)		
Anthracycline based	4 (5)		
Taxane based	41 (51)		
Vinorelbine	15 (18.5)		
Capecitabine	6 (7)		
Trastuzumab plus endocrine therapy	4 (5)		
	
*Duration of previous trastuzumab (weeks)*			
Median (range)	39 weeks (5 weeks–38 months)		
<12	4		
12–24	16		
>24	61		
	
*Treatment at progression*			
Trastuzumab alone	11 (13.5)		
Trastuzumab plus chemotherapy	44 (54)		
Taxane based	9 (11)		
Capecitabine	21 (26)		
Vinorelbine	14 (17)		
Trastuzumab plus endocrine therapy	2 (2.5)		
Trastuzumab plus radiotherapy	24 (30)		

Abbreviations: CNS=central nervous system; ER=oestrogen receptor; HER2=human epidermal growth factor receptor 2; MBC=metastatic breast cancer; PgR=progesterone receptor.

**Table 3:** Efficacy data for the 114 patients with HER2+ve locally advanced or MBC (response rate, TTP and OS)

	**No. of patients (%)**
*Radiological response*	77 (68)
Stable disease or better	55 (48)
Progressive disease	22 (20)
	
*Clinical response*	16 (14)
Stable disease or better	12 (11)
Progressive disease	4 (3)
	
Median duration of trastuzumab (95% CI)	10 months (8–11)
Median TTP (95% CI)	24 weeks (21–28)
Median OS (95% CI)	19 months (15–24)

Abbreviations: CI=confidence interval; HER2=human epidermal growth factor receptor 2; MBC=metastatic breast cancer; OS=overall survival; TTP=time to progression.

**Table 4:** Efficacy data for the 81 patients with HER2+ve locally advanced or MBC (response rate, TTP and OS)

	**No. of patients (%)**
*Radiological response*	54 (66)
Stable disease or better	40 (49)
Progressive disease	14 (17)
	
*Clinical response*	12 (15)
Stable disease or better	10 (12)
Progressive disease	2 (3)
	
Median duration of trastuzumab (95% CI)	11 months (9–14)
Median TTP (95% CI)	25 weeks (18–33)
Median OS (95% CI)	22 months (17–27)

Abbreviations: CI=confidence interval; HER2=human epidermal growth factor receptor 2; MBC=metastatic breast cancer; OS=overall survival; TTP=time to progression.
